# Cyclodextrin-Templated Co(II) Grids: Symmetry Control over Supramolecular Topology and Magnetic Properties

**DOI:** 10.1021/acs.inorgchem.1c03344

**Published:** 2022-01-24

**Authors:** Arkadiusz Kornowicz, Michał Terlecki, Iwona Justyniak, Daniel Prochowicz, Jan van Leusen, Paul Kögerler, Janusz Lewiński

**Affiliations:** † Institute of Physical Chemistry, Polish Academy of Sciences, Kasprzaka 44/52, 01-224 Warsaw, Poland; ‡ Faculty of Chemistry,Warsaw University of Technology, Noakowskiego 3, 00-664 Warsaw, Poland; § Institute of Inorganic Chemistry, RWTH Aachen University, Landoltweg 1, D-52074 Aachen, Germany

## Abstract

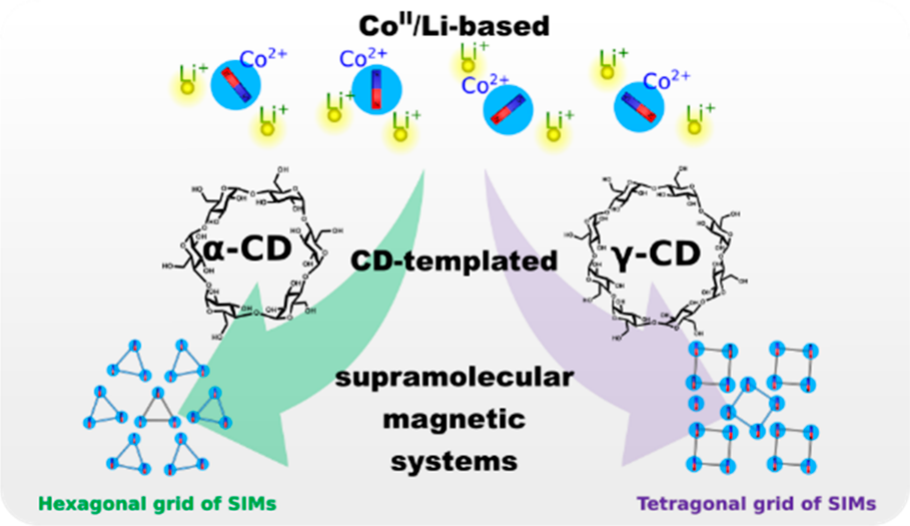

While inherent complexation properties and propensity for self-organization of cyclodextrins (CDs) render them potentially promising scaffolds of magnetic materials, this research area is still at an embryonic stage. We report on the synthesis and structure characterization of a new sandwich-type complex, [(α-CD)_2_Co_3_Li_6_(H_2_O)_9_] (**α-1**), which represents a smaller analogue of the previously characterized [(γ-CD)_2_Co_4_Li_8_(H_2_O)_12_] (**γ-1**) cluster. A comprehensive structural analysis of **α-1** and a careful reinvestigation of **γ-1** reveal how the symmetry of CD ligands determines the molecular composition and supramolecular arrangements of Co/Li sandwich-type complexes. Furthermore, the first comparative studies of the magnetic properties in this type of system point to subtle differences in the magnetic behavior of both compounds. The sandwich-type complexes **α-1** and **γ-1** exhibit field-induced slow magnetic relaxation, defining a new family of magnetic materials with a pillared grid-like supramolecular structure composed of weakly interacting Co^II^ centers forming an SMM.

## Abstract 2

Cyclodextrin-based coordination systems are potentially promising scaffolds of supramolecular materials, including functional magnetic systems. A comprehensive structural analysis of α- and γ-cyclodextrin-based Co(II) coordination complexes reveals how the symmetry of macrocyclic ligands determines the molecular composition and supramolecular arrangements of Co/Li sandwich-type structures. Furthermore, the first comparative studies of the magnetic properties in this type of system point to subtle differences in the magnetic behavior of both compounds.

## Introduction

Naturally occurring cyclodextrins (CDs) are readily available macrocyclic entities with an inherent hydrophobic internal cavity and hydrophilic external surface that display a combination of interesting molecular recognition and complexation properties.^1^ These inherent properties identify CDs as very attractive host templates in the development of novel host–guest systems as well as molecular metal complexes and efficient building units in the construction of hybrid supramolecular materials with a desired functionality and prospective applications in catalysis, sensing, materials science, and medicine. The first structurally well-characterized CD–metal complex, [(β-CD)_2_Cu_4_Li_7_(H_2_O)_7_], was reported by Klüfers in 1993.^2^ Unfortunately, because of the challenges associated with the isolation of well-defined systems and/or reliable characterization methods, the interactions of native cyclodextrins with metal ions have remained a largely undeveloped research area, and the scarcity of structural data and mechanistic insights are some of the key obstacles in the rational design of new CD-based functional systems.^1c^ Nevertheless, comprehensive analysis of reported CD–metal complexes indicates that there are several distinguishable patterns of CD–metal interactions, which may be harnessed to control the chemical environment and spatial arrangement of metal centers.^1c^ In particular, native CDs possess a tendency for stabilization of sandwich-type metal complexes composed of macrocyclic systems of metal ions enclosed between two CD ligands.^1c^ Usually, heterometallic metallamacrocycle systems with incorporated auxiliary alkali metal ions are formed, although a few examples of homometallic CD-based sandwich-type complexes are also known, such as [(γ-CD)_2_Pb_16_]·20H_2_O.^1c,3^ In the former case, the rim size of the utilized CD and the type of auxiliary ions determine the composition of the resulting heterometallic macrocycles. Especially interesting is the effect of the auxiliary ions, where the utilization of Li^+^ ions usually provided {M,Li,Li}_*n*_-type metallamacrocycles, while Na^+^ results in an alternating {M,Na}_*n*_-type systems. Notably, the tendency of CD-based complexes for supramolecular self-organization via numerous cooperative hydrogen bonds provides an additional level of tailorability in the spatial packing of metal centers.

All the above-mentioned characteristics of CD-based coordination systems make them potentially promising scaffolds of supramolecular magnetic materials; nevertheless, this research area still is at an embryonic stage. To the best of our knowledge, there are only two reports describing the detailed magnetic characterization of well-defined CD-based metal complexes. In 2009 Oshio and co-workers reported a sandwich-type β-CD complex, [Na_7_[(V=O)_7_Na_7_(H_2_O)_7_(β-CD)_2_]·65H_2_O, containing a heterometallic metallamacrocycle composed of seven magnetically active vanadyl (VO^2+^) ions separated by the auxiliary Na^+^ ions.^4^ The distances between vanadyl ions turned out to be short enough (ca. 6.2–6.4 Å) to allow for significant antiferromagnetic coupling between the seven spin 1/2 vanadyl groups. This resulted in two nearly degenerate *S* = 1/2 spin ground states, affected by the ring distortions. More recently, we utilized the coordination properties of γ-CD ligands for the rational synthesis of a heterometallic Co^II^ complex [(γ-CD)_2_Co_4_Li_8_(H_2_O)_12_] with the individual magnetic centers separated by two Li^+^ ions resulting in Co···Co distances of about 10.7–10.9 Å.^5^ This provided a rather good magnetic isolation between the individual Co^II^ ions, which exhibit a field-induced slow magnetic relaxation consistent with the single ion magnet (SIM) behavior, or the system of a grid of four Co^II^ centers forms a single-molecule magnet (SMM), which is replicated within the supramolecular architecture.

As part of our continuing research on the design and synthesis of functional materials based on CD building units^5,6^ and homo- and heterometallic clusters incorporating magnetically active metal ions^7^ herein, we demonstrate how the type of CD ligand influences the formation, self-organization, and magnetic properties of Co/Li sandwich-type complexes. To this effect, we have isolated and structurally characterized a new sandwich-type complex, [(α-CD)_2_Co_3_Li_6_(H_2_O)_9_], which represents a smaller macrocycle analogue of the [(γ-CD)_2_Co_4_Li_8_(H_2_O)_12_] cluster. The comparison of α-CD and γ-CD derivatives shows how the symmetry of CD ligands determine the molecular composition and supramolecular arrangements of Co/Li sandwich-type complexes and influence the magnetic separation of Co^II^ centers.

## Experimental Section

### Synthetic Materials and Methods

α-Cyclodextrin (α-CD), β-cyclodextrin (β-CD), and γ-cyclodextrin (γ-CD) were purchased from Cavamax W8 Pharma. Commercially available (Sigma-Aldrich) cobalt chloride hexahydrate and lithium hydroxide monohydrate were used as received without further purification. Elemental analyses were performed on Elementar VarioMicro Cube analyzer, and FTIR spectra were recorded on Bruker TENSOR II spectrometer by using the ATR technique. ICP-OES measurements were performed by the Central Institute for Engineering, Electronics and Analytics (ZEA-3), Forschungszentrum Jülich GmbH (D-52425 Jülich, Germany), on a Thermo Scientific iCAP6500 spectrometer featuring an Echelle polychromator, a CID detector, axial and radial view torch, and wavelength coverage of 166–847 nm. The following procedure was performed twice: 50 mg of the sample was dissolved in a mixture of 3 mL of HNO_3_ and 3 mL of H_2_O_2_, which was filled up to a volume of 50 mL, of which two aliquots were diluted in a ratio of 1:100 and analyzed.

#### Synthesis of [(α-CD)_2_Co_3_Li_6_(H_2_O)_9_] (**α-1**)

α-CD (243 mg, 0.25 mmol) and hydrated cobalt(II) chloride, CoCl_2_·6H_2_O (119 mg, 0.5 mmol), were dissolved in 2 mL of H_2_O and slowly dropped at room temperature to monohydrated lithium hydroxide, LiOH·H_2_O (524 mg, 12.5 mmol), and α-CD (240 mg, 0.247 mmol) suspended in 2 mL of water. After few minutes of mixing, the resulting deep violet-blue solution was filtered and carefully introduced to the vapor of acetone. Pink-violet needle-like crystals formed within 2 weeks and were collected by filtration (yield 318 mg). Results of the elemental analysis of the bulk material may vary due to difficulties with purification of the crude material from lithium salts residues. The amount of Co^2+^ and Li^+^ ions in the bulk material used in magnetic studies was determined by ICP-OES; found: Co 6.3%, Li 2.63%. Elemental analysis found: C 32.00%, H 5.91%, O 50.90%. Based on these results, the resulting heterometallic complex may be formulated as [(α-CD)_2_Co_3_Li_6_(H_2_O)_9_]·24.15(H_2_O)·2.42(LiOH)·1.84(LiCl) (Co 6.5%, Li 2.62%, C 31.75%, H 5.83%, O 50.91%) (*M*_w_ = 2721.09 g/mol). FTIR (ATR): ν = 3311 (w), 2916 (w), 2363 (vw), 2109 (vw), 1984 (vw), 1622 (w), 1428 (m), 1361 (m), 1296 (w), 1151 (m), 1082 (m), 1005 (s), 949 (m), 862 (m), 747 (m), 710 (m), 476 (s) cm^–1^.

#### Synthesis of β-CD Analogue

A suspension of β-CD (243 mg, 0.215 mmol) hydrated cobalt(II) chloride, CoCl_2_·6H_2_O (119 mg, 0.5 mmol), in 2 mL of H_2_O was slowly added at room temperature to monohydrated lithium hydroxide, LiOH·H_2_O (524 mg, 12.5 mmol) and β-CD (240 mg, 0.247 mmol) suspended in 2 mL of water. After a few minutes of mixing, a resulting deep violet-blue solution was filtered and carefully introduced to the vapor of acetone. Many attempts to obtain high-quality single crystals resulted in violet thread-like precipitate contaminated by a brownish sludge. Results of the elemental analysis of the bulk material were diverging and inconsistent.

#### Synthesis of [(γ-CD)_2_Co_4_Li_8_(H_2_O)_12_] (**γ-1**)

The complex was synthesized according to the previously reported procedure.^5^ Pink-violet needle-like crystals of **γ-1** were collected by filtration after crystallization by diffusion of acetone vapor into the parent solution. Results of the elemental analysis of the bulk material may vary due to difficulties with purification of the crude material from lithium salts residues. The amount of Co^2+^ and Li^+^ ions in the bulk material used in magnetic studies was determined by ICP-OES; found: Co 6.49%, Li 1.86%. Elemental analysis found: C 31.30%, H 6.20%, O 49.80%. Based on these results, the resulting heterometallic complex may be formulated as [(γ-CD)_2_Co_4_Li_8_(H_2_O)_12_]·34.56(H_2_O)·3.58(LiCl) (Co 6.4%, Li 2.21%, C 31.46%, H 6.37%, O 50.06%) (*M*_w_ = 3661.63 g/mol). FTIR (ATR): ν = 3311 (w), 2916 (w), 2104 (vw), 1987 (vw), 1613 (w), 1478 (m), 1428 (s), 1359 (w), 1154 (m), 1083 (m), 1000 (s), 942 (m), 863 (m), 756 (m), 709 (m), 483 (s), 404 (w) cm^–1^.

### Single-Crystal X-ray Diffraction

The crystals were selected under Paratone-N oil, mounted on the nylon loops and positioned in the cold stream on the diffractometer. The X-ray data for complexes **α-1** and **γ-1** were collected at 100(2)K on a SuperNova Agilent diffractometer using graphite monochromated Mo Kα radiation (λ = 0.71073 Å). The data were processed with CrysAlisPro.^8^ The structure was solved by direct methods using the SHELXS-97 program and was refined by full matrix least-squares on *F*^2^ using the program SHELXL.^9^ All non-hydrogen atoms were refined with anisotropic displacement parameters. Hydrogen atoms were added to the structure model at geometrically idealized coordinates and refined as riding atoms.

#### Crystal Data for **α-1**

C_216_H_282_Co_9_Li_30_O_339_: *M* = 9040.97 g/mol, trigonal, space group *P*321 (no. 150), *a* = 29.3000(5) Å, *b* = 29.3000(9) Å, *c* = 15.8540(10) Å, *U* = 11787.0(8) Å^3^, *Z* = 1, *F*(000) = 4623, *D*_c_ = 1.274 g cm^–3^, μ(Mo Kα) = 0.423 mm^–1^, θ_max_ = 24.697°, 13351 unique reflections. Refinement converged at *R*1 *=* 0.1001, *wR*2 = 0.2264 for all data, 908 parameters, and 21 restraints (*R*1 = 0.0906, *wR*2 = 0.2193 for 11757 reflections with *I*_0_ > 2σ(*I*_0_)). The goodness-of-fit on *F*^2^ was equal 1.091.

#### Crystal Data for **γ-1**

C_96_H_128_Co_4_Li_8_O_116_: *M* = 3429.22, tetragonal, space group *P*4 (no. 75), *a* = 25.4682(9) Å, *b* = 25.4682(9) Å, *c* = 15.3628(6) Å, *U* = 9964.8(8) Å^3^, *Z* = 2, *F*(000) = 3528, *D*_c_ = 1.143 g cm^–3^, μ(Mo Kα) = 0.422 mm^–1^, θ_max_ = 26.497°, 20315 unique reflections. Refinement converged at *R*1 *=* 0.1672, *wR*2 = 0.2258 for all data, 1015 parameters and 7 restraints (*R*1 = 0.0917, *wR*2 = 0.1928 for 8351 reflections with *I*_0_ > 2σ(*I*_0_)). The goodness-of-fit on *F*^2^ was equal 0.889.

### Magnetic Measurements

The magnetic data of **α-1** and **γ-1** were collected by using a Quantum Design MPMS-5XL SQUID magnetometer. The polycrystalline samples were compacted and immobilized into cylindrical PTFE capsules. The data were recorded as a function of the magnetic field (0.1–5.0 T at 2.0 K) and the temperature (2–290 K at 0.1 T) and were corrected for the diamagnetic contributions of the sample holder and the compound (χ_m,dia_/10^–3^ cm^3^ mol^–1^ = −1.67 (**α-1**), −1.82 (**γ-1**)). In addition, dynamic (ac) susceptibility measurements were performed in the ranges 2–50 K and 3–1000 Hz at zero and 700 Oe static bias magnetic field by using an amplitude of *B*_ac_ = 3 × 10^–4^ T.

## Results and Discussion

### Synthesis and Structure Characterization

#### Synthesis

**Figure 1:**
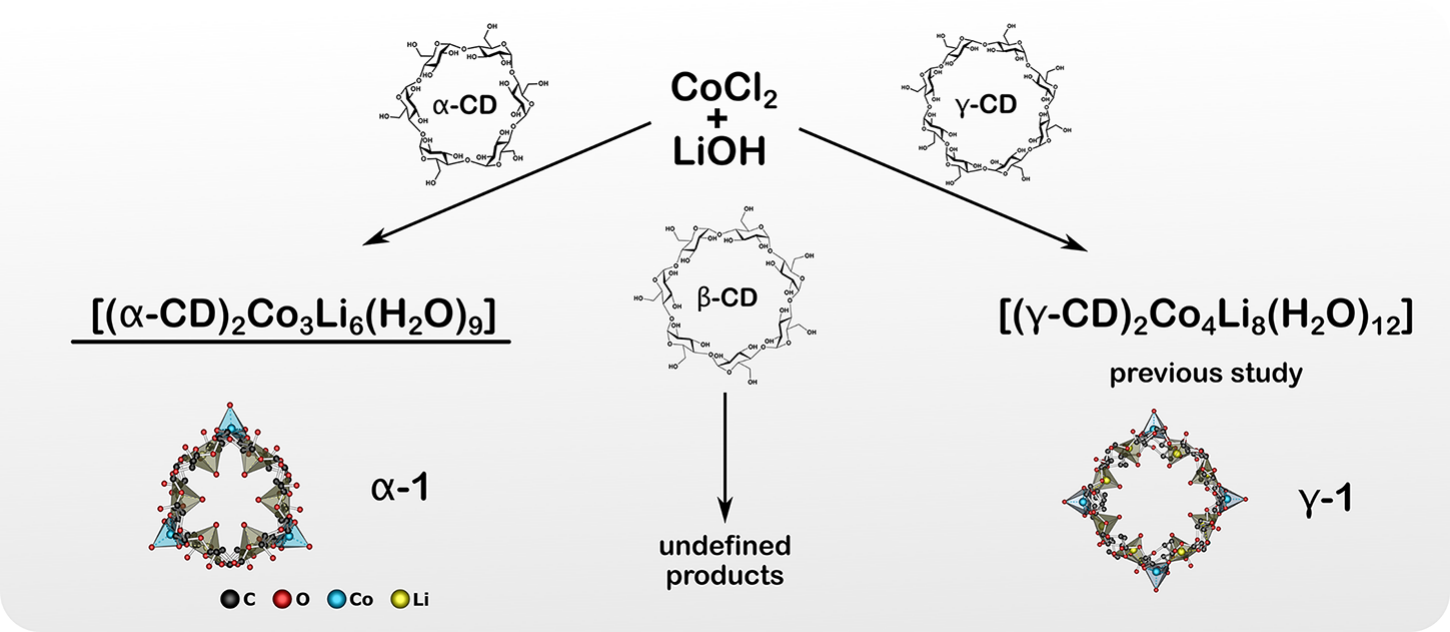
Scheme of the synthesis of sandwich-type heterometallic Co/Li complexes stabilized by various CD ligands.

A new heterometallic Co/Li complex [(α-CD)_2_Co_3_Li_6_(H_2_O)_9_] (**α-1**) incorporating two α-CD anions was obtained by a procedure previously reported for [(γ-CD)_2_Co_4_Li_8_(H_2_O)_12_] (**γ-1**)^5^ (Figure 1) utilizing optimized amounts of reactants (see the Experimental Section). To this aim, an aqueous solution of α-CD and CoCl_2_ was slowly dropped to LiOH and α-CD suspended in water. Well-formed pink needle-like crystals of **α-1** were isolated from the parent solution by slow diffusion of acetone vapor. Compound **α-1** is insoluble in common organic solvents and decomposes in nonalkaline aqueous solutions. The [(α-CD)_2_Co_3_Li_6_(H_2_O)_9_]·24.15(H_2_O)·2.42(LiOH)·1.84(LiCl) stoichiometry of the resulting crystalline material was estimated by using ICP-OES and elemental analysis. Additionally, the product was characterized by single-crystal and powder X-ray diffraction (SCXRD and PXRD, respectively) and FTIR spectroscopy along with magnetometry analysis of the dc and ac susceptibility. A similar reaction with β-CD resulted in an undefined mixture of products. Numerous attempts to obtain good-quality single crystals from the reaction mixture usually resulted only in a violet thread-like precipitate contaminated by a brownish sludge. The difficulties in the formation of well-defined product in this case are probably a result of mismatched symmetry between β-CD ligands and {Co,Li,Li}_*n*_-type metallamacrocycles. In fact, to the best of our knowledge, there are only two known examples of heterometallic sandwich-type complexes of β-CD: one with asymmetric {Cu,Li,Li}_3_{Cu,Li} metallamacrocycle, which is based on square-planar Cu centers,^2^ and one with alternating {V,Na}_7_ metallamacrocycle characteristic for systems templated by Na^+^ ions.^4^

Furthermore, for a more in-depth understanding of the templating effect of CD ligands on the stoichiometry of the resulting heterometallic complexes as well as their molecular and supramolecular structure and magnetic properties, we repeated the synthesis and characterization of [(γ-CD)_2_Co_4_Li_8_(H_2_O)_12_] (**γ-1**) complex under identical conditions. Then we performed a comparative study of the structure and magnetic properties of compounds **α-1** and **γ-1**. Single-crystal X-ray analysis of the newly synthesized **γ-1** showed that its molecular and crystal structure are in line with that reported previously (*vide infra*).^5^ The stoichiometry of the resulting crystalline material of **γ-1** was identified as [(γ-CD)_2_Co_4_Li_8_(H_2_O)_12_]·34.56(H_2_O)·3.58(LiCl) via SCXRD, ICP-OES, and elemental analysis (see the Experimental Section).

#### Molecular Structure

**Figure 2:**
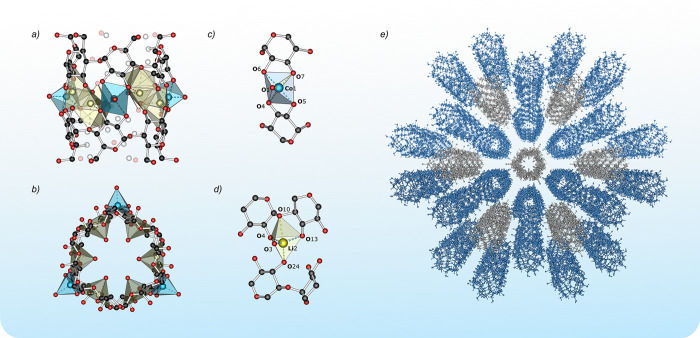
Structure of **α-1**: top (a) and side (b) view on the molecular structure; coordination sphere of the Co^2+^ (c) and Li^+^ (d) ions; pillar-like supramolecular structure (e).

**Figure 3:**
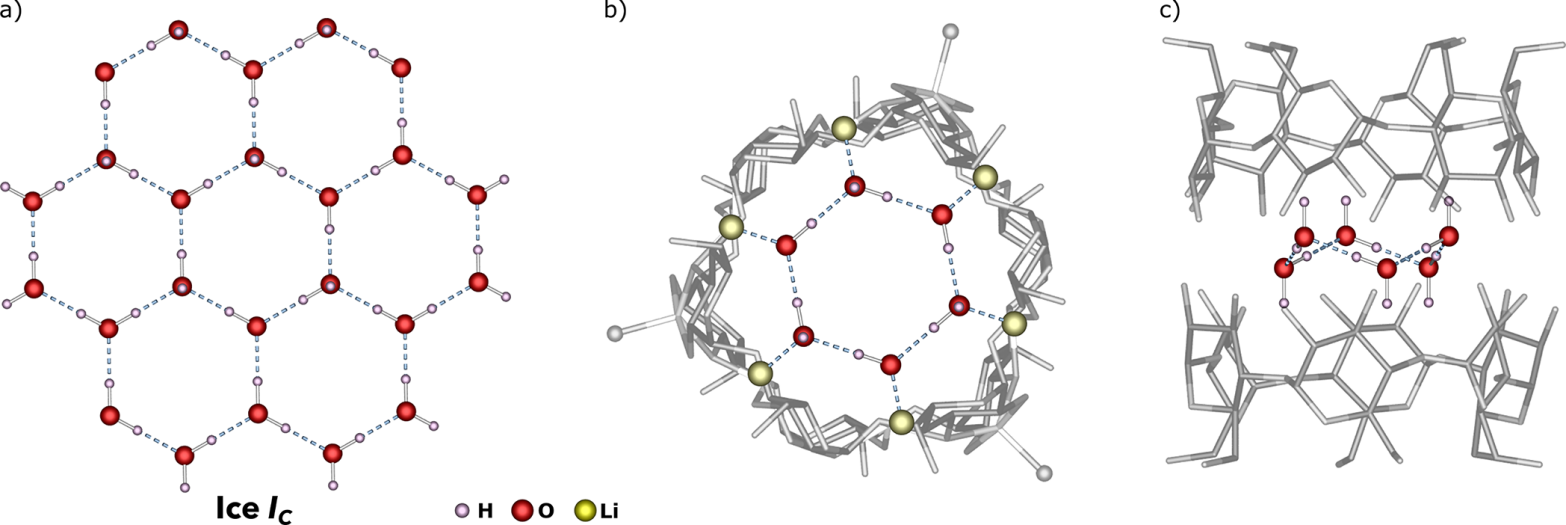
Supramolecular layer of water molecules in the crystal structure of cubic ice *I*_*c*_ (a) and top (b) and side (c) view on the homodromic hexagonal (H_2_O)_6_ aggregate inside the **α-1** molecules.

The crystal structure of **α-1** comprises two crystallographically independent molecules of the sandwich-type complex [(α-CD)_2_Co_3_Li_6_(H_2_O)_9_]. Each of them consists of a nona-membered heterometallic {Co,Li,Li}_3_ ring confined by partially deprotonated α-CD ligands (Figure 2). The metallamacrocycle contains three Co^II^ and six Li^+^ ions grouped in three {Co,Li,Li} units in a triangular arrangement. Each of the two crystallographically independent Co^II^ centers has a similar CoO_5_ coordination environment with a distorted trigonal-bipyramidal geometry, one of them, Co2, with additional crystallographically imposed *C*_2_ symmetry (Table 1). Four oxygen atoms in the Co^II^ coordination sphere come from the two CD ligands (two from the alkoxide and two from the secondary hydroxyl groups), while the fifth oxygen atom belongs to the coordinated water molecule directed outward the ring skeleton. The Li^+^ centers adopt a distorted trigonal-bipyramidal geometry of the coordination sphere composed of two alkoxide, one ether, and one hydroxyl oxygen atoms of the two CD ligands, and the additionally coordinated water molecule. In contrast to the Co-bonded water molecules, the Li-bonded water molecules are pointed inside the metallamacrocycle forming a hydrogen-bonded hexameric aggregates (O–O distances: 2.646–2.724 Å; O–O–O angles: 103.4°–104.3°) with a chairlike conformation, which resembles the basic building units of cubic ice *I*_*c*_ (Figure 3). The formation of similar hexametric water molecule aggregates inside sandwich-type α-CD complexes was previously noticed by Klüfers and co-workers in the heterometallic systems incorporating {Fe^II^,Li,Li}_3_, {Mn^II^,Li,Li}_3_, {(V^IV^O),Na}_6_, and {Bi^III^/Na}_6_-type matallamacrocycles.^10^ In this view, the interior of the barrel-shaped α-CD-based heterometallic complexes ensures a proper environment for the homodromic hexagonal (H_2_O)_6_ aggregates providing a unique supramolecular support for this type structure (Figure 3).

**Table 1:** Analysis of the Coordination Sphere Geometry of Co^II^ Centers in **α-1** and **γ-1** Using the Continuous Shape Measurement (CShM) and the Geometry Index τ_5_

	SHAPE (CShM)^a^		
metal center	trigonal bipyramid	square pyramid	geometry index (τ_5_)^b^
**α-1** Co1	0.77	2.99	0.71
**α-1** Co2	0.98	2.70	0.66
**γ-1** Co1	0.83	2.94	0.69
**γ-1** Co2	0.63	3.07	0.72

a Lower values indicate better fit to given geometry.^11^

b Distinguish whether the geometry of the coordination sphere is trigonal bipyramidal (close to 1) or square pyramidal (close to 0).^12^

The molecular structure of **γ-1** represents a larger sandwich-type analogue of **α-1** and comprises the metallamacrocycle composed of four {Co,Li,Li} units in a square geometry.^5^ The coordination environment of the metal centers is similar in both complexes (Table 1), and the analogous separation of Co^II^ centers by pairs of Li^+^ ions provides comparable intramolecular Co···Co distances of about 10.3 and 10.7 Å, respectively. The Li-bonded water molecules in the interior of **γ-1** are relatively separated (O···O distances: 2.880–3.605 Å) and on their own do not form any similar homodromic aggregates like in the smaller interior of **α-1**.

#### Supramolecular Structure

**Figure 4:**
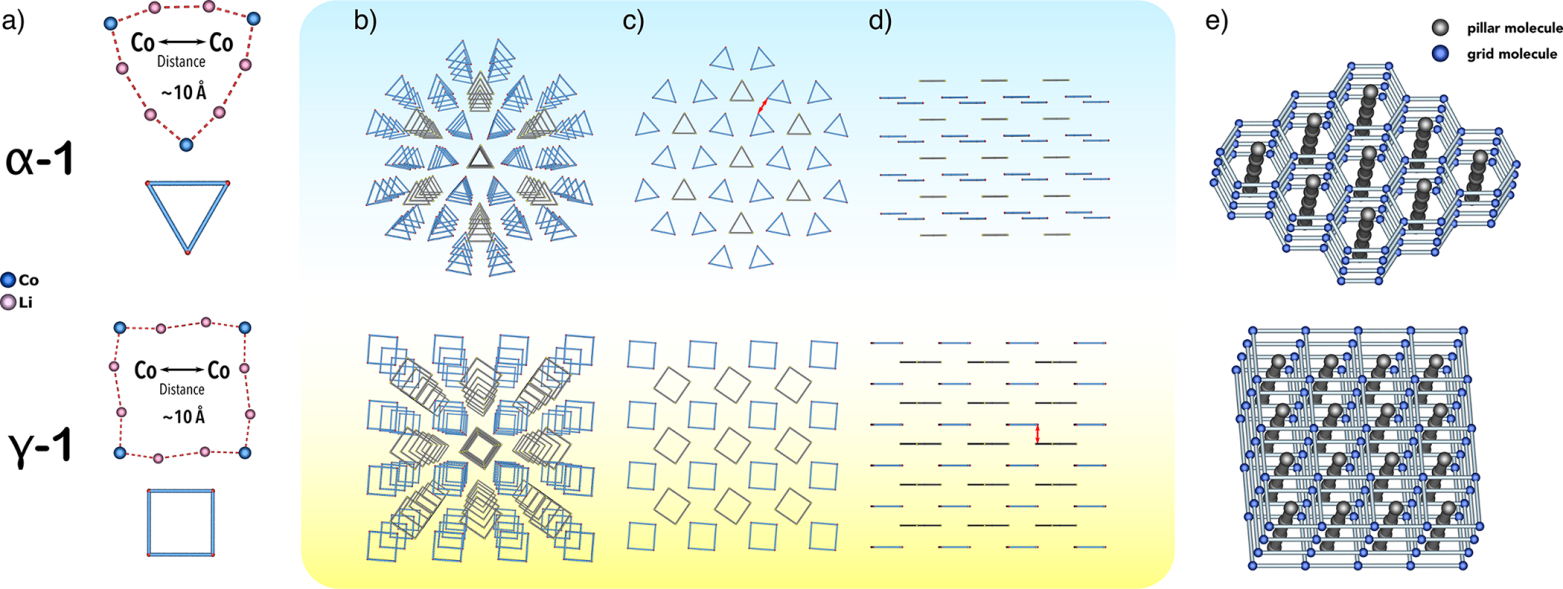
Comparison of the supramolecular architectures of **α-1** and **γ-1**: the composition and geometry of the heterometallic macrocycles (a), perspective (b), top (c), and side (d) views of the supramolecular structures (a representative of the shortest intermolecular Co···Co distance is marked in red); schematic representations of the pillared grid-like frameworks (e).

Complexes **α-1** and **γ-1** crystallize in the trigonal *P*321 and tetragonal *P*4 space group, respectively, with two essentially identical symmetrically independent sandwich-type molecules in the unit cell. In the crystal lattice **α-1** molecules self-assemble into a pillared grid-like supramolecular structure with 1D open channels along the *c*-axis (Figure 2e). Two types of the symmetrically independent **α-1** molecules form alternating supramolecular layers, where one of them is arranged into a honeycomb-like grid deformed by a slight differentiation in the altitude of molecules within individual layers (Figures 2e and 4, blue molecules), and the second acts as pillars filling gaps between the hexagonal grids (Figures 2e and 4, gray molecules). As we demonstrated previously, molecules of **γ-1** form a supramolecular structure with a 4-fold symmetry.^5^ In this case, the grid-type layers have a square geometry (Figure 4, blue molecules), which determines the similar arrangement of the molecules in the pillar layers filling the grid gaps (Figure 4, gray molecules). The observed differences in the geometry of the 2D layers in the supramolecular structures of **α-1** and **γ-1** affect the spatial separation of the Co^II^ ions. In the grid layers of **α-1** the closest intermolecular Co···Co distances are about 8.2 Å, which is significantly shorter than the respective distances of about 10.7 Å in **γ-1**. Furthermore, in **α-1**, the pillar molecules are arranged in a way that favors close intermolecular Co–Co distances (i.e., vertex-to-vertex), while in **γ-1**, the pillar molecules are twisted by an angle of 35° compared to the molecules in the grid layers (i.e., vertex-to-edge), which favors a more effective supramolecular separation of the Co^II^ centers between the molecules in neighboring layers. Nevertheless, in both supramolecular structures, the shortest intermolecular Co–Co distances with the pillar molecules have a comparable value of 9.2 and 9.6 Å for **α-1** and **γ-1**, respectively. Interestingly, while both α-CD and γ-CD act as effective template agents for heterometallic Co/Li systems with pillared grid-like supramolecular architecture, the shortest Co···Co distances in **α-1** are about 8.2 Å and are localized between the molecules in the honeycomb-like layers, while in **γ-1** the shortest Co···Co distances are significantly longer at about 9.6 Å and localized between the molecules in neighboring supramolecular layers (Figure 4c,d).

A more in-depth crystal structure analysis reveals that both compounds **α-1** and **γ-1** cocrystallize with a significant amount of water molecules entangled in hydrogen-bonding networks within the intermolecular regions. Moreover, the X-ray analysis of **α-1** indicates the presence of additional tetrahydrated Li^+^ ions integrated within the product crystals during the crystallization (three Li^+^ per one **α-1**, see Figure S2). Similar incorporation of Li^+^ ion impurities is not observed in the crystal structure of **γ-1**. The number of co-included water molecules and Li^+^ ions estimated from ICP-OES and elemental analysis is about 24 and 34 H_2_O, and about 4 and 3 Li^+^ per one molecule of **α-1** and **γ-1**, respectively (*vide supra*), which is significantly more than what is found in the crystal structure analysis. This divergence is likely related to impurities occluding the crude macrocrystalline materials. Interestingly, the larger barrel-shaped molecules of **γ-1** exhibit more dense packing in the crystal lattice compared to the smaller **α-1** analogue. The calculated solvent accessible voids are 5956.6 and 4520.4 Å^3^, which are 50.5 and 45.4% of the unit cell volume for **α-1** and **γ-1**, respectively, which are consistent with the larger numbers of the co-included exterior H_2_O molecules and Li^+^ ions in the α-CD derivative.

### Magnetic Properties

The selection of organic ligands used for the stabilization of the metal–organic system plays a crucial role in the development of magnetic materials like single-molecule magnets (SMMs)^13^ or single-ion magnets (SIMs).^14^ Utilization of per-design ligands enables the control over physicochemical properties of the magnetic systems by influencing on (i) magnetic anisotropy of metal centers through governing their primary coordination sphere,^15^ (ii) magnetic interaction between metal centers by the construction of chemical bridges between them,^16^ and (iii) spatial distribution of magnetic centers via supramolecular self-assembly.^17^ Unfortunately, the knowledge in this field is still limited, and a more sophisticated understanding of the influence of stabilizing ligands on magnetic properties of metal centers is necessary for the rational designing of magnetic systems. The above structural analysis of **α-1** and **γ-1** nicely showcases how the geometry of CD ligands may efficiently dictate the spatial arrangement and separation of Co(II) centers in the crystal lattice. Thus, to gain a more in-depth understanding of the structure–magnetic properties relationship in CD-templated sandwich-type complexes, we performed a detailed magnetic characterization of both materials.

**Figure 5:**
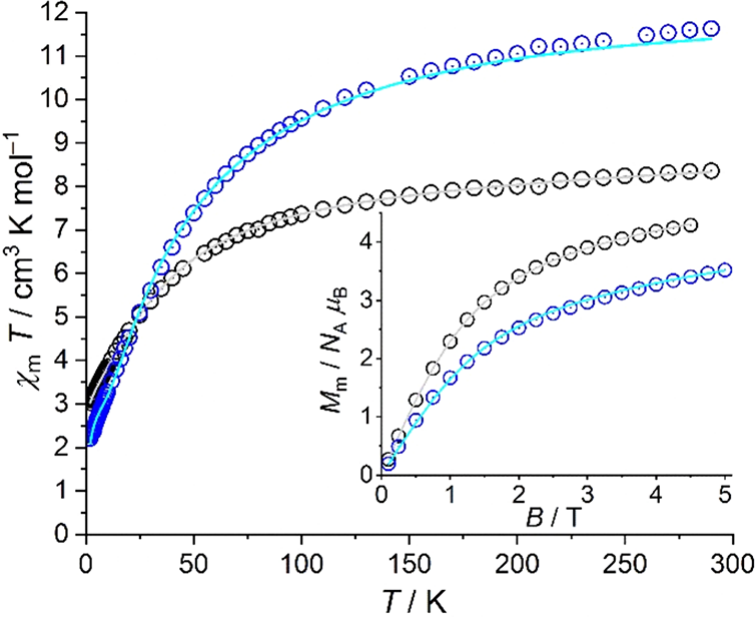
Magnetic dc measurements: χ_m_*T* vs temperature *T* at 0.1 T and (inset) molar magnetization *M*_m_ vs magnetic field *B* at 2.0 K of **α-1** (black symbols) and **γ-1** (blue circles). Solid lines represent least-squares fits.

The magnetic properties of **α-1** and **γ-1** in a static magnetic field are shown in Figure 5 as well as Figures S3 and S4 as χ_m_*T* vs *T* plots at 0.1 T, *M*_m_ vs *B* plots at 2.0 K, and χ_m_ vs *T* plots at 0.1 T, respectively. For **α-1**, the value of χ_m_*T* is 8.36 cm^3^ K mol^–1^ at 290 K, which is within the expected^18^ range 6.94–10.14 cm^3^ K mol^–1^ for three noninteracting high-spin Co^II^ centers. Upon cooling, the values of χ_m_*T* gradually decrease with temperature and rapidly decrease at *T* < 100 K, reaching 3.03 cm^3^ K mol^–1^ at 2.0 K. The molar magnetization at 2.0 K is approximately linear up to 1 T and noticeably flattens at higher fields, reaching 4.3 *N*_A_ μ_B_ at 4.5 T. Most, if not all (considering the Co···Co distances), of the χ_m_*T* value decrease is due to the thermal depopulation of the energy states originating from the free ion Russell–Saunders term ^4^*F*. This term is split into ^4^A_2_″, ^4^A_1_′, ^4^A_2_′, ^4^E′, and ^4^E″ terms by a trigonal-bipyramidal ligand field (*D*_3*h*_), which are further split into Kramers doublets by the ligand field of actual lower symmetry and spin–orbit coupling. The latter also introduces mixing with excited terms, in particular with the ^4^A_2_″ and ^4^E′ terms arising from the ^4^P term. However, the χ_m_*T* value at 2.0 K is remarkably low as is the value of *M*_m_ of about 4–5 *N*_A_μ_B_. Such a value usually indicates only two noninteracting high-spin Co^II^ centers. Before discussing this observation, we analyze the magnetic properties of **γ-1**.

The χ_m_*T* value of **γ-1** is 11.63 cm^3^ K mol^–1^ at 0.1 T and 290 K being within the expected^19^ range 9.25–13.53 cm^3^ K mol^–1^ for four noninteracting high-spin Co^II^ centers. Upon cooling, the values of χ_m_*T* continuously decrease with a steeper slope at *T* < 150 K reaching 2.21 cm^3^ K mol^–1^ at 2.0 K. At this temperature, the molar magnetization is linear up to fields of ca. 1 T. At higher fields, the magnetization slowly increases up to 3.5 *N*_A_ μ_B_ at 5.0 T. Besides the reasons already mentioned in the case of **α-1** for this behavior, the magnitudes at 2.0 K are smaller for **γ-1** than for **α-1**, even though there is an additional Co^II^ center in the structure of the former. Therefore, weak exchange interactions are most likely present in the compounds although the Co–Co distances are rather large. Because the behavior of the curves, χ_m_*T* vs *T* and *M*_m_ vs *B*, is rather similar to that of higher dimensional systems as for example chains of paramagnetic centers, the exchange interactions seem to be intermolecular and antiferromagnetic, although intramolecular interactions cannot be definitely excluded. A higher dimensional, weakly interacting magnetic system can be justified from the structural information. We thus developed a magnetochemical model to reproduce these observations by the concurrent fitting of the combined susceptibility and magnetization data by using the CONDON framework.^19^ Implementing a “full model” approach that is ideally geared toward transition metal spin centers, CONDON considers all 120 energy microstates of a 3d^7^ valence electron configuration for each Co^II^ center as well as interelectronic repulsion and spin–orbit coupling.

The exchange interactions are modeled by a mean-field approach represented by the parameters *z*(*−*2*J*), i.e., considering *z* nearest-neighbor centers and using the “*–*2*J*” notation for the Heisenberg–Dirac–van Vleck-type exchange interactions. We assume the three Co^II^ centers in **α-1** to be identical, as we do for the four centers of **γ-1**, but different in both compounds, since there are small yet significant differences in their local geometries. The *D*_3*h*_ ligand field symmetry is fully represented by the two ligand field parameters *B*^2^_0_ and *B*^4^_0_. However, the corresponding fits are of inadequate quality, and we thus investigated the structural information in terms of the point charge electrostatic model. According to these PCEM results, the ligand field is better represented as distorted *D*_3*h*_ with an overlaying *C*_2*v*_ symmetry, characterized by dominant contributions in *B*^2^_0_ and *B*^4^_0_ and secondary contributions in *B*^2^_2_, *B*^4^_2_, and *B*^4^_4_. Adopting this local symmetry situation, we identify parameters that yield a very high fit quality, characterized by a low *SQ* (relative root-mean-square error) value (Table 2). The corresponding χ_m_*T* vs *T* curves are shown as blue and gray solid lines in Figure 5. The found ligand field parameters are rather similar in both compounds (with the exception of *B*^2^_2_). The exchange interactions in both compounds are weak and predominantly antiferromagnetic. Even though the number of closest neighbors *z* is larger in **γ-1** compared to **α-1**, they do not differ by an order of magnitude, as do the *z*(*−*2*J*) values. Therefore, albeit relatively weak, the exchange interactions are stronger in **γ-1** than in **α-1**. The lowest Kramers doublet energies of the single Co^II^ centers in **α-1** are 57.4 cm^–1^ for the first and 153.0 cm^–1^ for the second excited doublet, relative to the ground state. Further parameters can be approximately determined, which are used in effective theories, such as the zero-field splitting parameters *D* and *E*. Note that even for the lowest energy states the results of such theories slightly differ from the results of the “full model”. This is due to the latter being more comprehensive and including usually more energy states often inducing mixing of states. For **α-1**, *D* ≈ −28 cm^–1^ and *E* ≈ 4 cm^–1^. In **γ-1**, the corresponding energies are 30.5 and 176.3 cm^–1^ for the first and second excited doublet, respectively, and *D* ≈ +14 cm^–1^ and *E* ≈ 3 cm^–1^. Finally, we note that it would be ideal to augment these model descriptions with EPR data, which however remained outside the scope of this study.

**Table 2:** Parameters of the Least-Squares Fits of the DC SQUID Magnetometry Data (in cm^–1^)^a^

	**α-1**	**γ-1**
*B*	1115	
*C*	4366	
ζ	533	
*B*^2^_0_	17890 ± 8	20428 ± 16
*B*^2^_2_	–11573 ± 6	–6251 ± 5
*B*^4^_0_	40480 ± 20	41787 ± 4
*B*^4^_2_	7067 ± 13	6462 ± 10
*B*^4^_4_	625 ± 9	820 ± 63
*z*(*−*2*J*)	–0.2 ± 0.1	–2.1 ± 0.1
*SQ*	0.6%	1.8%

a Ligand field parameters *B*^*k*^_*q*_ in Wybourne notation; Racah parameters *B*, *C*, and one electron spin–orbit coupling constant ζ taken from ref (24).

**Figure 6:**
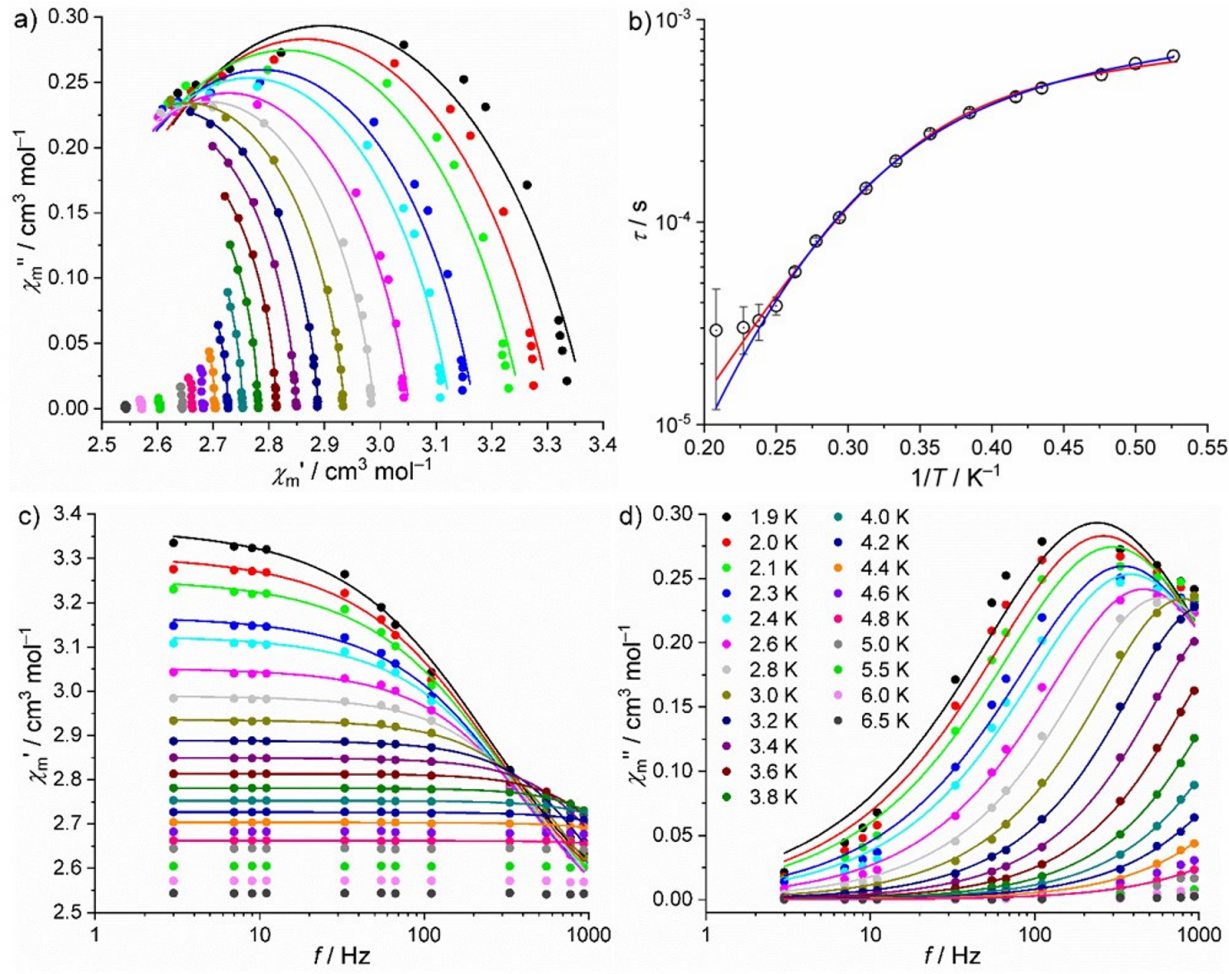
Magnetic ac measurements of **α-1** at 700 Oe static bias field: (a) Cole–Cole plot; (b) Arrhenius plot of relaxation times τ vs 1/*T*; (c) in-phase component of magnetic ac susceptibility χ_m_′ vs frequency *f*; (d) out-of-phase component of magnetic ac susceptibility χ_m_″ vs *f* (symbols: data; lines: least-squares fits (a, c, d) to generalized Debye expression; (b) to an Orbach and a direct (red line, model A) or to a Raman and direct (blue line, model B) slow relaxation process).

The response of **α-1** and **γ-1** in a dynamic magnetic field did not show any significant out-of-phase signals, i.e., no relevant slow relaxation processes, at zero static bias field. However, adjusting the static bias field to 700 Oe shows such signals. In the case of **α-1**, the corresponding data are shown in Figure 6. Distinct out-of-phase signals are detected up to 5.0 K and analyzed in terms of the generalized Debye expression^20^ by simultaneously fitting χ_m_′ vs *f* and χ_m_″ vs *f* at each measurement temperature. The corresponding least-squares fits yield the solid lines shown in Figure 6a,c,d as well as the relaxation times τ shown in Figure 6b as open symbols. The distribution of the relaxation times α = 0.133 ± 0.098 suggests few relaxation pathways, since it is close to yet significantly larger than zero. We therefore consider as potential processes quantum tunneling of magnetization (QTM), Orbach, Raman, and direct relaxation processes. While a direct relaxation process is definitely present, the distinction between Orbach or Raman process as additional contribution cannot be unambiguously determined from the data, which was also noted elsewhere for similar compounds.^5,21^ Because a satisfactory and sound correlation between the energy states of the paramagnetic centers and the parameters deduced from magnetic ac measurements data could still not be derived, partially due to the models including (virtual) phonon processes, we present both results that have almost the exact same fit quality. For an Orbach and a direct relaxation process, the relation is τ^–1^ = τ_0_^–1^ × exp(−*U*_eff_/*k*_B_*T*) + *A*_K_*T* (model A), while for Raman and direct processes this equation reads τ^–1^ = *CT*^*n*^ + *A*_K_*T* (model B). Note that *A*_K_ is a function of the applied field *H*. The order is either *H*^2^ or *H*^4^ depending on whether hyperfine interaction or the electrostatic potential, respectively, dominates the direct relaxation process.^22^ The results are an attempt time τ_0_ = (9.68 ± 1.71) × 10^–8^ s, an effective energy barrier *U*_eff_ = 17.4 ± 0.5 cm^–1^, and *A*_K_ = 841 ± 19 K^–1^ s^–1^ (model A) or *C* = 1.08 ± 0.28 K^–*n*^ s^–1^, *n* = 7.1 ± 0.3, and *A*_K_ = 753 ± 25 K^–1^ s^–1^ (model B) at 700 Oe static bias field. The quality of the least-squares fit is marginally better in the case of model A; however, the parameters *A*_K_ of both models are remarkably large. However, upon consideration of the approximately derived effective model parameters *D* and *E* from the dc data analysis, these indicate an eas*y*-axis system, which favors an Orbach slow relaxation process. The order *n* of the Raman process is different from 9 (or 5), i.e., the values commonly observed for Kramers systems, however, such a value is possible if certain criteria are met at the measured temperature range.^23^

**Figure 7:**
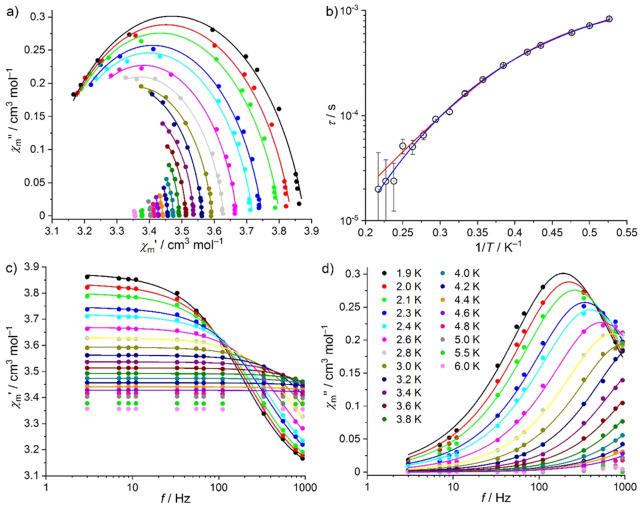
Magnetic ac measurements of **γ-1** at 700 Oe static bias field: (a) Cole–Cole plot; (b) Arrhenius plot of relaxation times τ vs 1/*T*; (c) in-phase component of magnetic ac susceptibility χ_m_′ vs frequency *f*; (d) out-of-phase component of magnetic ac susceptibility χ_m_″ vs *f* (symbols: data; lines: least-squares fits (a, c, d) to generalized Debye expression; (b) to an Orbach and a direct (red line, model A) or to a Raman and direct (blue line, model B) slow relaxation process).

The magnetic ac susceptibility data of **γ-1** are shown in Figure 7. Relevant out-of-phase signals are detected up to 4.6 K. The analysis in terms of the generalized Debye expression yields the solid lines shown in Figure 7a,c,d and the relaxation times τ shown in Figure 7b. The distribution of the relaxation times α = 0.117 ± 0.052 suggests few relaxation pathways. By considering as potential processes QTM, Orbach, Raman, and direct relaxation processes, we are confronted by the same observations as in the analysis of the **α-1** data: The distinction between an Orbach or a Raman process (in addition to the direct relaxation process) cannot be unambiguously determined. We, thus, present both resulting sets of fit parameters. The parameters employing model A are an attempt time τ_0_ = (6.61 ± 0.14) × 10^–7^ s, an effective energy barrier *U*_eff_ = 12.0 ± 0.5 cm^–1^, and *A*_K_ = 566 ± 19 K^–1^ s^–1^. Employing model B, we find *C* = 12.5 ± 2.6 K^–*n*^ s^–1^, *n* = 5.5 ± 0.2, and *A*_K_ = 424 ± 26 K^–1^ s^–1^. Both data sets were taken at 700 Oe static bias field. The quality of the least-squares fit is slightly better by using model B. However, the parameters *A*_K_ of both models are large, and the order *n* of the Raman process is different from 9 yet closer to 5.

To emphasize, the application of a small static bias field is necessary to reveal slow relaxation in both compounds. The dominant process is the by this means induced direct relaxation process. In comparison to similar compounds,^5,22^ the parameters for the Orbach relaxation processes are similar, while the Raman and the direct process parameters are noticeably different, which may be due to the potential exchange interactions suggested by the magnetic dc susceptibility data. We note that the applied mean-field approach does not take into account interactions between spins and the lattice, while all considered slow relaxation processes are spin–lattice interactions. Based on this model, this could be an indirect effect since only the individual spin contributions may be affected.

## Conclusions

The reported studies nicely substantiate that native cyclodextrins are favorable compounds in formulating small molecule clusters and exiting supramolecular structures as well as in developing single-molecule magnet systems. On this occasion, we demonstrate that both α-CD and γ-CD act as effective template agents for heterometallic sandwich-type Co/Li systems with a similar pillared grid-like supramolecular architecture, which provide efficient spatial separation between the magnetic centers. The size and composition of the Li-templated {M,Li,Li}_*n*_ metallamacrocycle confined within sandwich-type complexes are determined by the type and symmetry of CD ligands. The utilization of α-CD and γ-CD results in the formation of systems with trigonal and tetragonal symmetry, **α-1** and **γ-1**, respectively, while in the case of β-CD we were not able to isolate any well-defined products, which is probably a result of mismatched symmetry of β-CD ligands and {Co,Li,Li}_*n*_-type metallamacrocycles. A comprehensive analysis of the crystal structures of **α-1** and **γ-1** reveals an interesting relationship between both the geometrical parameters of these molecular complexes and the topology of the supramolecular lattice. Specifically, both complexes exhibit a similar pattern of self-assembly into the similar pillared grid-like supramolecular architecture, where the geometry of the 2D supramolecular grid-like layers is dictated by the molecular symmetry of the sandwich-type complex. This difference in the supramolecular structure of **α-1** and **γ-1** affects the spatial separation between the Co^II^ centers. The shortest Co–Co distances in **α-1** are about 8.2 Å and localized between the molecules within the same 2D supramolecular grid-like layer. In turn, the shortest Co–Co distances in **γ-1** are significantly longer, about 9.6 Å, and located between the molecules from the neighbor supramolecular grid-like layers. Both compounds exhibit the field-induced slow magnetic relaxation characteristic for Co^II^-based SMMs.^15b,23^ The determined parameters for the Orbach relaxation processes are in line with similar compounds, whereas the Raman and the direct process parameters are different, which may be a result of the potential exchange interactions suggested by the magnetic dc susceptibility data. Interestingly, the magnitudes of the molar magnetization at 2.0 K are smaller for **γ-1** than that for **α-1**, even though there is an additional Co^II^ center in the structure of the former, which we tentatively attribute to the odd and even composition of antiferromagnetic metallamacrocycles in the molecular structures of **α-1** and **γ-1**, respectively, or the formation of higher dimensional, weakly interacting magnetic systems.

In conclusion, we demonstrated the high control over the spatial distribution of metal centers at molecular and supramolecular levels provided by the CD ligands, which substantiates their high potential as promising scaffolds of functional materials. Furthermore, although CDs have been successfully used for the stabilization of molecular,^4^ supramolecular,^5^ and nanometric^24^ magnetic systems, to the best of our knowledge, we presented the first comparative studies of the magnetic properties in the CD-templated systems. The results provide a promising starting point for further studies, which we believe will contribute to a more in-depth understanding of the structure–magnetic properties relationship, which is crucial for the development of magnetic materials.

## Supporting Information Available

Experimental and simulated PXRD patterns, arrangement of Li^+^ ions in the crystal lattice of **α-1**, magnetic dc susceptibility measurements scaled to a single Co(II) center and molar magnetic susceptibility χ_m_ vs temperature *T* (PDF)

## Accession Codes

CCDC 2035406–2035407 contain the supplementary crystallographic data for this paper. These data can be obtained free of charge via www.ccdc.cam.ac.uk/data_request/cif, or by emailing data_request@ccdc.cam.ac.uk, or by contacting The Cambridge Crystallographic Data Centre, 12 Union Road, Cambridge CB2 1EZ, UK; fax: +44 1223 336033.
