# Supplementary material for: Cyclodextrin-Templated Co(II) Grids: Symmetry Control over Supramolecular Topology and Magnetic Properties
Source: Inorg Chem. 2022 Jan 24;61(5):2499–508. doi: 10.1021/acs.inorgchem.1c03344 (PMC8826275; doi:10.1021/acs.inorgchem.1c03344)
Supplement: Supplementary file 1 — ic1c03344_si_001.pdf [file ic1c03344_si_001.pdf]

Supporting Information

Cyclodextrin-templated Co(II) grids: Symmetry control over supramolecular topology and magnetic properties

Arkadiusz Kornowicz,[†] *Michał Terlecki,*[‡] *Iwona Justyniak,*[†] *Daniel Prochowicz,*[†] *Jan van Leusen,*[§] *Paul Kögerler**[,§] *and Janusz Lewiński**[,†,‡]

[†]Institute of Physical Chemistry, Polish Academy of Sciences, Kasprzaka 44/52, 01-224 Warsaw (Poland)

[‡]Faculty of Chemistry,Warsaw University of Technology, Noakowskiego 3, 00-664 Warsaw (Poland)

[§]Institute of Inorganic Chemistry, RWTH Aachen University, Landoltweg 1, D-52074 Aachen (Germany)

*E-mail: lewin@ch.pw.edu.pl
*E-mail: paul.koegerler@ac.rwth-aachen.de

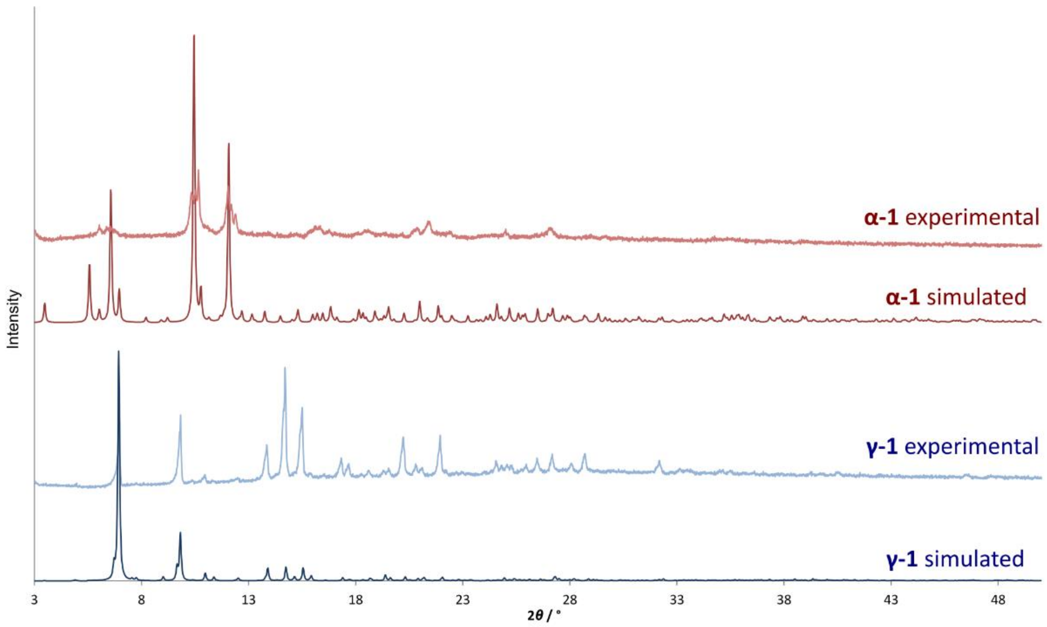

Figure S1. Experimental and simulated PXRD patterns of **α-1** and **γ-1**

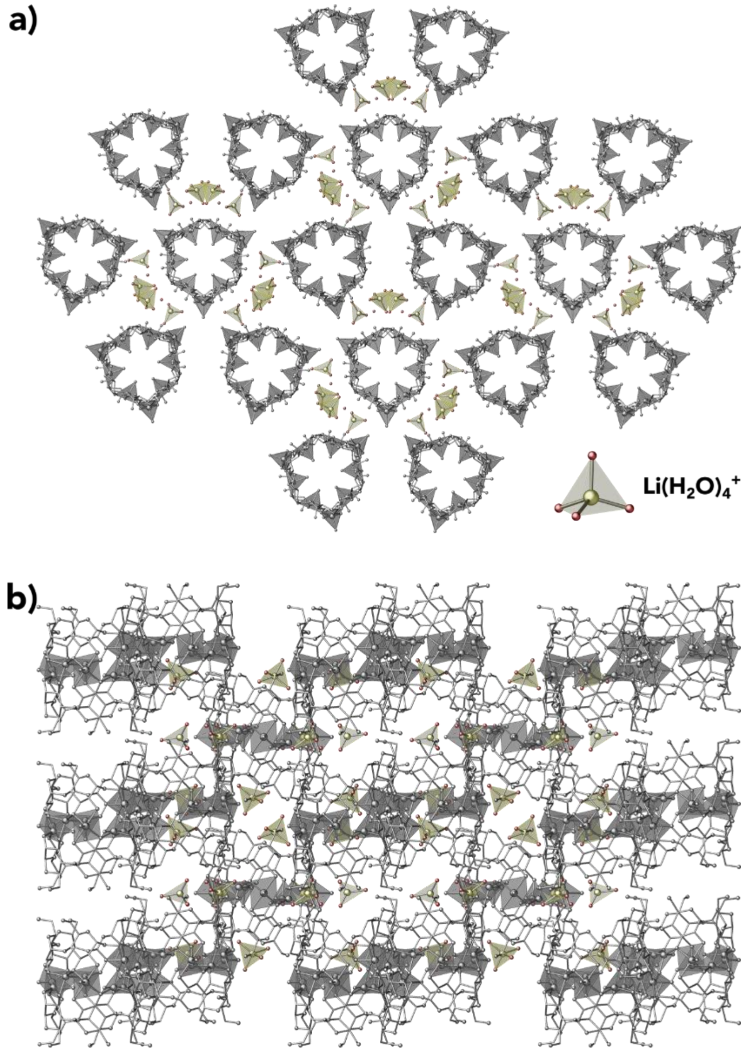

Figure S2. Arrangement of Li^+ ions in the crystal lattice of **α-1.**

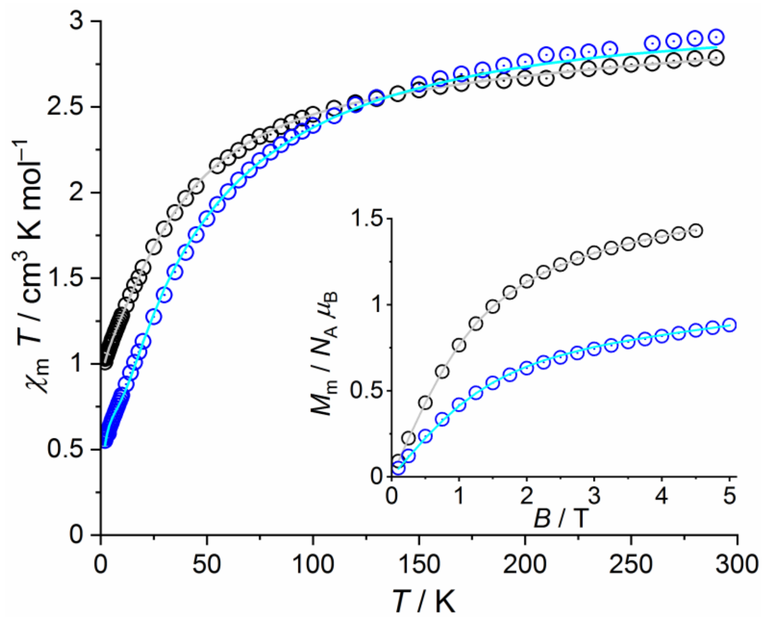

Figure S3. Magnetic dc susceptibility measurements: $\chi_m T$ vs. T and M_m vs. B plots of **α-1** and **γ-1** as shown in Figure 5, however, with data scaled to a single Co(II) center per formula unit.

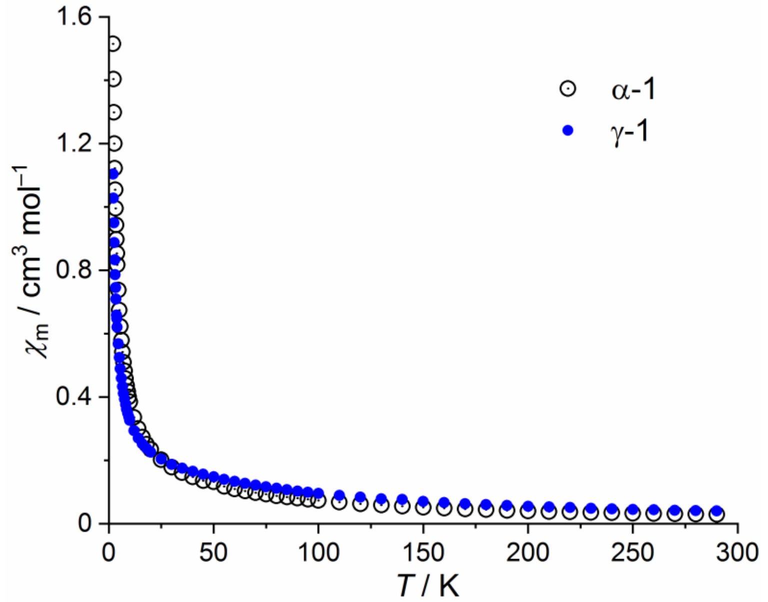

Figure S4. Molar magnetic susceptibility χ_m vs. temperature T at 0.1 T for **α-1** (black open circles) and **γ-1** (blue full circles).
